# Extracorporeal photopheresis—New insights into an old procedure

**DOI:** 10.1111/tme.13156

**Published:** 2025-06-10

**Authors:** Sabine Seiffert, Janine Kirchberg, Enrica Bach, Victoria Menger, Mandy Brückner, Ulrich Sack, Ulrike Köhl, Uwe Platzbecker, Andreas Boldt, Marco Herling, Vladan Vučinić

**Affiliations:** ^1^ Institute of Clinical Immunology University Leipzig Medical Center Leipzig Germany; ^2^ Department of Hematology, Cellular Therapy, Hemostaseology, and Infectious Diseases University Leipzig Medical Center Leipzig Germany; ^3^ Comprehensive Cancer Center Central Germany (CCCG) Leipzig Germany; ^4^ Fraunhofer Institute for Cellular Therapies Leipzig Germany

**Keywords:** apoptosis, ECP, T‐cell proliferation

## Abstract

**Background:**

Extracorporeal photopheresis (ECP) is a safe immunomodulatory strategy that induces cell‐type selective apoptosis through photodynamic processes. Despite decades of use, the mechanisms underlying ECP remain largely unexplored, particularly in studies examining specific immune cell subsets in ex vivo setups.

**Aims:**

This proof‐of‐concept pilot study presents data on apoptosis and proliferation of T‐lymphocytes following ex vivo ECP application to leukocyte concentrates (LC) and peripheral blood (PB) samples from healthy donors.

**Methods:**

LC and PB were diluted to a haematocrit of 2% and treated with 8‐methoxypsoralen, followed by ECP (ECP+) or no ECP (ECP−) in a discontinued system. Apoptosis of mononuclear cells was assessed 48 h post‐ECP using annexin V and 7 Aminoactinomycin D (7‐AAD) staining with flow‐cytometric quantification. The proliferative capacity of non‐apoptotic T‐lymphocytes was measured after 72 h of post‐ECP stimulation with anti‐CD3/CD28 cross‐linking, using Violet Proliferation Dye 450.

**Results:**

ECP exposure significantly reduced the median T‐cell receptor‐induced proliferation of viable T‐lymphocytes from both LC (4.6%, *p* = 0.02) and PB (4.2%, *p* = 0.03). However, 7‐AAD staining 48 h post‐ECP showed no significant differences in the proportions of apoptotic cells in this experimental model.

**Conclusion:**

Ex vivo ECP treatment inhibited T‐lymphocyte proliferation in both LC and PB from healthy individuals, suggesting this as a key mode of action. Our findings highlight ECP's potential applications, including its implications for modern immune therapies' adverse effects. Further analyses of functional characteristics of remaining vital cells are necessary.

## 1 INTRODUCTION

Extracorporeal photopheresis (ECP) is an immunomodulatory leukapheresis‐based procedure with a firm place in the treatment armamentarium of patients with cutaneous T‐cell lymphoma (CTCL), graft‐vs‐host disease (GvHD) and rejection after solid organ transplantation.^1^, ^2^ During ECP, leukocytes enriched for mononuclear cells (MNC) are collected, incubated with the photosensitizer 8‐methoxypsoralen (8‐MOP) and exposed to ultra‐violet A (UVA) irradiation before reinfusion to the patient.^3^ UVA‐activated 8‐MOP causes cross‐links with cellular macromolecules, especially DNA, which, a few hours after reinfusion, results in lymphocyte apoptosis, which in turn modulates dendritic cells (DC).^4^ The final net effect is a complex immunomodulation, particularly of the T‐cell compartment, with an overall shift towards immune tolerance.^5^, ^6^

Monocytes undergo apoptosis after ECP, as well. As monocytes do not proliferate, their apoptosis is slower and reduced compared to lymphocytes.^7^ Furthermore, after contact with extracorporeal surfaces, monocytes become activated and differentiate into immature DC, which eventually become loaded with patient‐specific antigens.^8^ The immature DCs perform phagocytosis of apoptotic lymphoid cells but also of early apoptotic monocytes. Subsequently, the DCs mediate a process called trans‐immunisation, that is, maturation and presentation of antigenic peptides.^6^ As a consequence, the balance between T helper (Th) cells 1 and 2 is shifted towards Th2 cells, resulting in the induction of anti‐inflammatory and reduction of pro‐inflammatory cytokines.^9^, ^10^

Since 1987, several closed and open ECP systems have been available for clinical use. In a closed ECP system (i.e. a ‘one‐step’ method), the steps of cell separation, drug photoactivation and re‐infusion are fully integrated and automated.^11^ Despite being used for several decades, some mechanisms of action of ECP remain speculative, while some have been revealed in more detail during recent years.^12^

There are published data on levels of induced apoptosis in lymphocytes from established cell lines at 24 and 48 h after ECP treatment, but with a focus on the influence of haematocrit and plasma concentration on apoptosis efficiency.^13^ Taverna et al. proposed that the variation of apoptosis of more than 15% (∆ apoptosis) in samples treated with or without ECP should be used as the measure of efficacy of the method in vivo.^14^

To provide further insights into the pathophysiology of the method, we performed a series of in‐vitro experiments to quantify the levels of apoptosis of MNC originating from lymphocyte concentrates (LC) or from peripheral blood (PB) of healthy donors (HD) as well as to determine the effect of ECP on the proliferative capacity of non‐apoptotic T‐lymphocytes.

## 2 MATERIALS AND METHODS

### 2.1 Starting material

Five commercially available LC were 1:1 diluted with human serum from male blood group AB clotted whole blood (Sigma‐Aldrich). The donors were aged between 50 and 60 years (*n* = 3) and under 30 years (*n* = 2). Additionally, naïve PB of four HD was analysed. The HDs were aged 30–40 years (*n* = 3) and 50–60 years (*n* = 1).

### 2.2 Preparation of LC and PB of HD

LC and PB were diluted with 0.9% NaCl in ECP treatment bags (Macogenic Set, Macopharma) to a haematocrit of 2%. After the addition of 8‐MOP (UVADEX™Therakos Europe Ltd) to a final concentration of 340 ng/mL 8‐MOP the samples were exposed to UV light at 2 J/cm^2^ using a Macogenic G2 device (Macopharma).

As negative controls, untreated diluted LCs and PB were utilised. Following ECP treatment, MNC of all ECP treated samples (ECP+) with associated untreated controls (ECP−) were isolated using density gradient centrifugation. Therefore, each sample was transferred and pelletised in 50 mL reaction tubes (800 × *g*, 10 min), resuspended in 35 mL Phosphate Buffer Saline (PBS) and subjected to density gradient (Pancoll human PAN Biotech) centrifugation (800 × *g*, 20 min). Interphase‐harvested MNC were washed twice and finally cultured at a concentration of 2 × 10^6^ cells/mL in Rosewell Park Memorial Institute Medium (RPMI), 20% human serum (Sigma‐Aldrich) and 1% penicillin/streptomycin (Gibco™) for 48 h at 37°C and 5% CO_2_ in 48‐well plates. The blood cell counts of LCs and PB prior to and after dilution are presented in Table 1.

**TABLE 1:** Median counts of treated cells, median percentage of vital, apoptotic and dead cells. Median counts of cells undergoing proliferation assay, proportion of proliferating cells.

Analysed cells	LC	PB
Starting material WBC count (/mL) (median, range)	**35 700** (30 500–53 700)	**6850** (5200–7700)
Cellcounts bevore cultivation (median, range) (×10^6^)		
ECP−	**12.0** (10.0–12.0)	**11.0** (7.0–10.0)
ECP+	**10** (10–12)	**9** (7–10)
Cellcounts after cultivation (median, range) (×10^6^)		
ECP−	**16.8** (6.0–52.0)	**21.2** (12.5–75.2)
ECP+	**10.2** (5.5–32.0)	**5.5** (3.9–13.6)
Vital cells (median, range) (%)		
ECP−	**87.9** (59.9–92.1)	**85.2** (81.3–89.1)
ECP+	**67.1** (60.6–71.4)	**72.4** (59.8–83.5)
*p* Value	**0.15**	**0.11**
Apoptotic cells (median, range) (%)		
ECP−	**5.3** (3.1–33.6)	**4.3** (3.6–10.6)
ECP+	**9.4** (7.9–21.5)	**4.3** (3.8–9.0)
*p* Value	**0.22**	**0.89**
Dead cells (median, range) (%)		
ECP−	**6.5** (5.3–10.4)	**10.9** (7.0–59.8)
ECP+	**20.6** (11.4–30.5)	**23.4** (12.6–31.2)
*p* Value	**0.08**	**0.49**
Vital T‐cells (median, range) (×10^6^/mL)		
ECP−	**40.2** (18.9–47.3)	**40.0** (10.7–68.5)
ECP+	**12.4** (3.8–15.3)	**17.2** (11.7–23.2)
*p* Value	** **0.01**	**0.49**
Proliferation (median, range) (%)		
ECP−	**49.2** (42.5–82.3)	**60.1** (44.8–69.8)
ECP+	**4.6** (0.0–47.4)	**4.2** (0.0–6.2)
*p* Value	* **0.02**	* **0.03**

Abbreviations: ECP, extracorporeal photopheresis; LC, leukocyte concentrates; PB, peripheral blood.

* statistically significant;

** statistically high significant.

### 2.3 Apoptosis assay

After 48 h incubation of both ECP+ and ECP− samples, approximately 0.5 × 10^6^ cultured MNC were transferred into fluorescence activated cell sorter (FACS) tubes and washed twice with cold Cell Staining Buffer (BioLegend®), resuspended in 100 μL Annexin V Binding Buffer (Biolegend®) and incubated for 15 min at room temperature (RT) in the dark with 5 μL of Annexin V and 5 μL of 7 Aminoactinomycin D (7‐AAD) (Biolegend®). To stop the reaction, 400 μL of Annexin V Binding Buffer (Biolegend®) was added to each tube. The percentage of viable and apoptotic MNC was recorded via flow‐cytometric measurements (BD FACSLyric™).^15^

### 2.4 Proliferation assay

**FIGURE 1:**
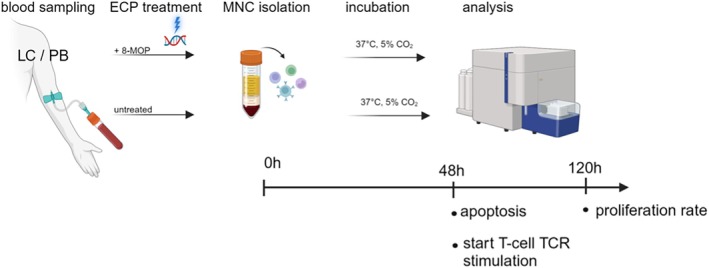
Apoptosis assays were performed on mononuclear cells (MNC) derived from leukocyte concentrates (LC) or peripheral blood (PB) of healthy donor following 48 h of post‐extracorporeal photopheresis (ECP) incubation (vs. no prior ECP treatment). Proliferation assays were performed after an additional 72 h of stimulation of non‐apoptotic T‐lymphocytes via anti‐CD3/CD28 cross‐linking. 8‐MOP, 8‐methoxypsoralen; TCR, T‐cell receptor.

Following the assessments of cell death, 0.25–1 × 10^7^ ECP treated cultured MNCs (ECP+) and corresponding negative controls (ECP−) were incubated with 1 μL 1 mM Violet Proliferation Dye 450 (violet proliferation dye [VPD]450) for 15 min and placed in a 37°C water bath. After incubation, cells were washed and counted in medium (RPMI 1640 [Gibco] including 10% fetal bovine serum (FBS) and 1% Pen/Strep). For each sample, wells in a 48‐well plate were prepared with 50 μL culture medium (RPMI 1640 [Gibco] including 10% FBS and 1% Pen/Strep) plus 50 μL anti‐Hu CD3/CD28 (clones: OKT3 and CD8.2, respectively [eBioscience™] for T‐cell receptor [TCR] stimulation [negative control with 100 μL culture medium only]). Finally, 0.5 × 10^6^ MNCs of each ECP+ and ECP− sample were added in each well at a volume of 500 μL and incubated for 72 h at 37°C in a 5% CO_2_ atmosphere. Afterwards, all samples were transferred to FACS tubes and washed twice with PBS 2 mM ethylenediaminetetraacetic acid (EDTA). Thereafter, anti‐CD3 (clone: SK7 [BD]) and anti‐CD45 (clone: 2D1 [BD]) were added to each tube and incubated for 15 min in the dark at RT. Finally, samples were washed with PBS 2 mM EDTA and 5 μL of 7‐AAD were added. After 15 min in the dark at RT, the reaction was stopped by adding 300 μL PBS 2 mM EDTA. Stained cells were analysed with BD FACSLyric™ as previously described.^15^ The procedures are depicted in Figure 1.

### 2.5 Statistics

Continuous variables were summarised as medians with ranges, while categorical variables were presented as frequencies and percentages. To analyse the effects of ECP treatment on MNC apoptosis and on the proliferative capacity of LC, the differences between the groups were tested using the Wilcoxon test. *p*‐Values <0.05 were assigned as significant. Estimations were performed with the R‐software (Version 4.3.1).^16^

## 3 RESULTS

### 3.1 Composition of initial cell materials

The median white blood cell (WBC) counts in LC samples were 35.7 (range 30.5–53.7) × 10^6^/mL and in PB 6.9 (5.2–7.7) × 10^6^/mL. Median 12 × 10^6^ MNC from LC underwent treatment with ECP− versus 10 × 10^6^ MNC in the ECP+ condition. In PB median MNC counts prior to ECP− and ECP+ treatment were 11 × 10^6^ and 9 × 10^6^, respectively.

### 3.2 ECP reduces viability of MNC

#### 3.2.1 Viability of MNC from LC

After 48 h of incubation, the median counts of MNC in LC in ECP− increased from 12.0 × 10^6^ (range 10.0–12.0 × 10^6^ cells) to 16.8 × 10^6^ (range 5.7–52.0 cells). In the ECP+ condition of LC samples, the median cell counts did not change and were 10.0 × 10^6^ (range 10.0–12.0) and 10.2 × 10^6^ (range 5.5–32.0), respectively. The median viability of ECP− versus ECP+ exposed MNC from LC was 88.0% (range 59.9–92.1) and 67.1% (range 60.6–71.4), respectively (*p* = 0.15).

The percentage of apoptotic cells in the ECP+ condition was higher than in the ECP−, but not statistically significant (*p* = 0.22). We noticed a trend for higher numbers of dead cells in the ECP+ versus ECP− with 6.5% (range 4.8–10.4) versus 20.6% (range 11.4–30.5), *p* = 0.08.

#### 3.2.2 Viability of MNCs from PB

After 48 h of culture, the median cell counts of MNC in the ECP− increased from 11.0 × 10^6^ (range 8.0–16.0) to 21.2 × 10^6^ (range 12.5–75.2) cells. In ECP+ condition, the median cell counts decreased from 9.0 × 10^6^ (range 7.0–10.0) to 5.5 × 10^6^ (range 3.9–13.6) cells.

**FIGURE 2:**
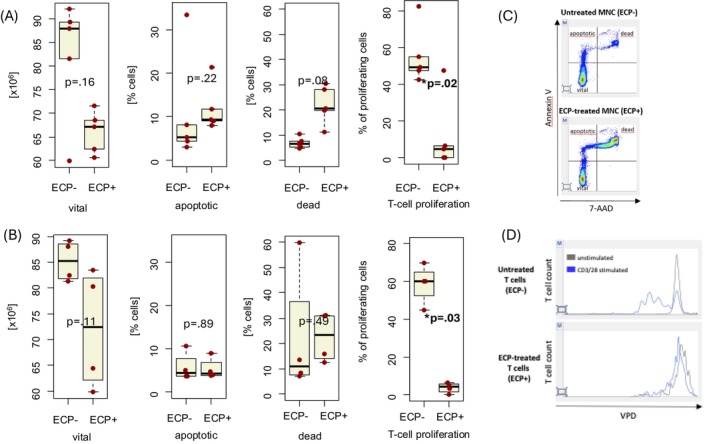
(A) Median counts of viable, apoptotic and dead mononuclear cells (MNC) and reduction of proliferation after extracorporeal photopheresis (ECP− and ECP+) in cells originating from leukocyte concentrates. (B) Median counts of viable, apoptotic and dead MNC and reduction of proliferation after ECP− and ECP+ in cells originating from peripheral blood. (C) Example of apoptosis testing per flow cytometry (Annexin V/7‐AAD staining). (D) Representative example of proliferation assay performed per assessment of dye dilution via flow cytometry (ECP inhibits CD3/CD28 mediated T‐cell proliferation). *statistically significant.

After ECP− versus ECP+ treatment, the median viabilities of MNC did not differ with 85.2% (range 81.3–89.1) versus 72.4% (range 59.8–83.5), respectively (*p* = 0.11) (Figure 2A,B).

There were no ECP‐associated differences regarding the number of apoptotic cells (*p* = 0.89) or dead cells (*p* = 0.49) in the PB samples. An example of the apoptosis test is presented in Figure 2C.

### 3.3 ECP reduces the induced proliferation of T‐lymphocytes derived from LCs and from PB

The median counts of viable cells after ECP− and ECP+ undergoing stimulated proliferation are presented in Table 1. After 72 h of culture of remaining viable cells of LC after ECP+ or ECP−, the median proportion of TCR‐induced proliferating cells (example presented in Figure 2D) was significantly higher in the ECP− condition than in the ECP+ with 49.2% (range 42.5–82.3) versus 4.6% (range 0–47.4), respectively (*p* = 0.02).

Similarly, the median proportion of TCR‐induced proliferating cells from PB after ECP+ was significantly lower with 4.2% (range 0.0–6.2) versus 60.2% (range 44.8–69.8) after ECP−, respectively (*p* = 0.03).

## 4 DISCUSSION

In view of a surprising shortage of ex vivo data, our pilot set of experiments demonstrated that the treatment with ECP induces apoptosis and inhibits the TCR‐mediated proliferative capacity of T‐lymphocytes, indicating potential modes of its overall immunomodulatory properties. Our in vitro data showed a median proportion of apoptosis of 9.4% in MNC from LC and of 4.3% in MNC from PB after ECP treatment. Our ∆ apoptosis is indeed lower when comparing this to reported data from Taverna et al. in a closed system, with most patients having ≥15% apoptosis 24 h after ECP treatment.^14^

Yoo et al. assessed the induction of apoptosis in a small population of CTCL patients and HD in an open system. They reported about apoptosis of nearly all cells 48 h after treatment with ECP in dosage 2–3 J UVA/cm^2^.^17^ Our data show a lower proportion of apoptotic cells, but a strong inhibition of proliferation in remaining 7‐AAD negative (viable) cells with only 4.6% of proliferating MNC from LCs and 4.2% from PB detected.

Open ECP systems use separate devices for cell separation and drug photoactivation (the ‘two‐step’ method). It is important to highlight that the combination of a device approved for separation and one for photoactivation is not equivalent to a closed system device approved for ECP. As several steps are involved in delivering therapy, there is a potential risk of infection, contamination, cross‐contamination and patient re‐infusion errors. In general, open systems can only be used by certified centres for handling blood components separately, whereas closed systems do not have such restrictions.^18^ Besides the small number of samples, one limitation of our experiments is that they were performed in a discontinued ECP System. Our data should be reevaluated in a system that is commonly used in current clinical settings, such as UVAR‐XTS® or CELLEX® (Therakos®).

A limitation of the present study is that the isolation of MNCs can effectively eliminate non‐viable cells. This procedure carries the risk of excluding biologically significant cellular populations present in the whole blood, which may result in an incomplete understanding of the cellular dynamics and interactions within the blood microenvironment.

Erythrocytes, for example, cannot be cultured, and therefore the analysis here was limited to lymphocytes to provide initial evidence of the mode of action of ECP. Future experiments will investigate the ECP effect on chimeric antigen receptor (CAR) T cells.

Nevertheless, our experiments provide insights into the dynamics of apoptosis and the inhibition of T‐lymphocyte proliferation after ECP treatment. It is important to differentiate between the quantitative inhibition of proliferation of T‐lymphocytes and functional impairment of their cytotoxicity. In ECP‐treated patients with GvHD, the apoptosis and consequent reduction of proliferation capacity of T‐lymphocytes do not impair their efficacy, that is, with respect to the graft‐vs‐leukaemia effect.^19^

Interesting is also the potential use of ECP in the context of evolving treatments with CAR T‐lymphocytes. In an experimental allogeneic CAR model, Han et al. demonstrated that ECP in the context of allogeneic CAR T‐cells could modulate their proliferative capacity but did not impair their long‐term functionality.^20^ One could hypothesise that ECP may have the potential to prevent or treat inflammatory complications following CAR T‐cell therapy.

More studies are needed to address this aspect of the mode of action of ECP, also beyond the usual indications like GvHD and CTCL.

## AUTHOR CONTRIBUTIONS

Conception and design: **Sabine Seiffert**, **Janine Kirchberg**, **Marco Herling** and **Vladan Vučinić**. Administrative support: **Ulrike Köhl**, **Uwe Platzbecker**. Provision of study materials: **Enrica Bach**, **Mandy Brückner**, **Ulrich Sack Andreas Boldt**. Collection and assembly of data: **Sabine Seiffert**, **Janine Kirchberg**, **Marco Herling**, **Victoria Menger**, **Vladan Vučinić**. Data analysis and interpretation: **Sabine Seiffert**, **Janine Kirchberg**, **Marco Herling**, **Victoria Menger**, **Vladan Vučinić**. Manuscript writing: All authors. Final approval of manuscript: All authors. Accountable for all aspects of the work: All authors.

## CONFLICT OF INTEREST STATEMENT

The authors have no competing interests.

## PATIENT CONSENT STATEMENT

Written informed consent was obtained from all individuals participating in the trial.

## Data Availability

Original data can be retrieved upon a reasonable request to the corresponding author.
